# Mitochondria-targeted antioxidant SkQ1 improves impaired dermal wound healing in old mice

**DOI:** 10.18632/aging.100772

**Published:** 2015-06-30

**Authors:** Ilya A. Demyanenko, Ekaterina N. Popova, Vlada V. Zakharova, Olga P. Ilyinskaya, Tamara V. Vasilieva, Valeria P. Romashchenko, Artem V. Fedorov, Vasily N. Manskikh, Maxim V. Skulachev, Roman A. Zinovkin, Olga Yu. Pletjushkina, Vladimir P. Skulachev, Boris V. Chernyak

**Affiliations:** ^1^ Faculty of Biology, Lomonosov Moscow State University, Moscow, Russia; ^2^ Belozersky Institute of Physico-Chemical Biology, Lomonosov Moscow State University, Moscow, Russia; ^3^ Institute of Mitoengineering, Lomonosov Moscow State University, Moscow, Russia; ^4^ Faculty of Bioengineering and Bioinformatics, Lomonosov Moscow State University, Moscow, Russia

**Keywords:** wound healing, mitochondrial reactive oxygen species, mitochondria-targeted antioxidant

## Abstract

The process of skin wound healing is delayed or impaired in aging animals. To investigate the possible role of mitochondrial reactive oxygen species (mtROS) in cutaneous wound healing of aged mice, we have applied the mitochondria-targeted antioxidant SkQ1. The SkQ1 treatment resulted in accelerated resolution of the inflammatory phase, formation of granulation tissue, vascularization and epithelization of the wounds. The wounds of SkQ1-treated mice contained increased amount of myofibroblasts which produce extracellular matrix proteins and growth factors mediating granulation tissue formation. This effect resembled SkQ1-induced differentiation of fibroblasts to myofibroblast, observed earlier *in vitro*. The Transforming Growth Factor beta (TGFβ)produced by SkQ1-treated fibroblasts was found to stimulated motility of endothelial cells *in vitro*, an effect which may underlie pro-angiogenic action of SkQ1 in the wounds. *In vitro* experiments showed that SkQ1 prevented decomposition of VE-cadherin containing contacts and following increase in permeability of endothelial cells monolayer, induced by pro-inflammatory cytokine TNF. Prevention of excessive reaction of endothelium to the pro-inflammatory cytokine(s) might account for anti-inflammatory effect of SkQ1. Our findings point to an important role of mtROS in pathogenesis of age-related chronic wounds.

## INTRODUCTION

Impaired wound healing represents a significant clinical problem in elderly. Pathogenesis of chronic wounds caused by diabetes mellitus, malnutrition, immunodeficiency, and aging is characterized by a prolonged self-sustaining inflammatory response, a defective fibroblast-dependent extracellular matrix (ECM) formation, and a failure of neovascularization and reepithelialization [1]. One of the common factors underlying compromised wound healing is excessive oxidative stress [2]. The leukocyte NADPH oxidase (Nox) is one of the major sources of ROS involved in pathogen killing [3], vascular endothelial growth factor (VEGF) signaling [4, 5], and TNF response [6]. Emerging evidence suggests that mitochondrial ROS (mtROS) are also important at various phases of the wound healing process. Recently it was reported that mtROS promoted actin-based closure of epithelial wounds in *Caenorhabditis elegans* [7]. In vertebrates, mtROS contribute to the anti-bacterial activity in macrophages [8]. Production of mtROS induced by TNF in endothelial cells [9] could be critical for the inflammatory response [10, 11]. VEGF-induced signaling that promotes endothelial cell migration also depends on mtROS [12]. On the other hand, excessive mtROS production resulting in decreased endothelial cell motility and inhibition of angiogenesis *in vivo* [13] may promote the aging skin phenotype [14]. Interestingly, expression of mitochondrial superoxide dismutase (MnSOD) in endothelial progenitor cells accelerates wound healing in diabetic mice [15].

Nevertheless, until now there was no evidence concerning involvement of mtROS in wound healing process of aging animals. The development of mitochondria-targeted antioxidants strongly facilitated the studies on the role of mtROS in normal and pathological processes. Mitochondria-targeted cationic derivates of coenzyme Q (MitoQ), vitamin E (MitoVitE) and SOD-mimetic TEMPO (MitoTEMPO) prevented cardiac dysfunction induced by ischemia-reperfusion, septic inflammation and endothelial dysfunction [7, 16-20]. *In vivo* experiments showed that mitochondria-targeted plastoquinones (SkQ1 and SkQR1) prevented nephropathy and brain damage induced by ischemia injury [21, 22], pielonephritis [23]. SkQ1 also retarded age-dependent development of osteoporosis, involution of thymus and spleen follicles, sexual activity in males [24]; myeloid shift in the blood and decline of estrous cycles in females [25, 26]; decline of exploratory behavior [27]; sarcopenia [28]; alopecia [26, 29]; retinopathy and cataract [24, 26, 29]; as well as increased the median lifespan of normal and progeric animals [25, 26, 29]. Thus, mitochondria-targeted antioxidants, at least partly fulfill requirements for the potential anti-aging drug [30].

Earlier we have found that scavenging of mtROS by SkQ1 *in vitro* led to the activation of TGFβ and the following myofibroblasts formation [31]. SkQ1 also stimulated *in vitro* wound closure in monolayers of fibroblasts and epitheliocytes [32]. These observations promoted our studies of SkQ1 in impaired wound models *in vivo*. In the present study, we have analyzed healing of dermal wounds in old mice.

## RESULTS

### SkQ1 stimulates granulation tissue formation and epithelization of the wounds

**Figure 1:**
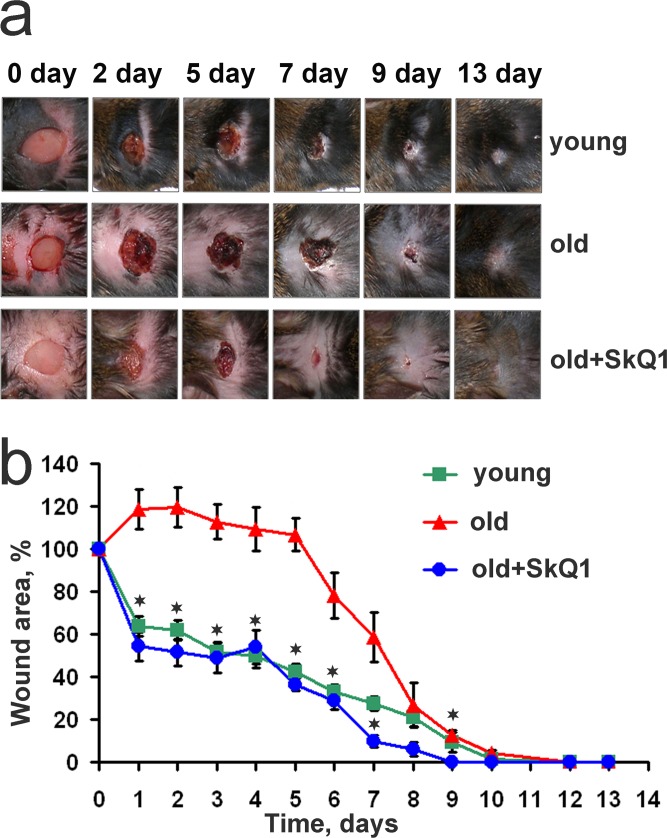
SkQ1 accelerates wound closure in old mice Full-thickness 7×7 mm excisions were made on the interscapular area of the back skin of young (6 month, n=12), old (24 month, n=12) and old mice received SkQ1 (100 nmol/kg of body weight per day) during the last 8 month of life (n=10). (**a**) Representative photos of wounds, (**b**) dynamics of wound closure. Data are presented as mean ± SD; *P < 0.05 for SkQ1-treated versus old mice.

In line with the previously published data [33, 34], old mice suffered from severely impaired wound healing. This effect was completely abolished in 24 month old animals treated with SkQ1 for last 8 months (Fig. 1).

**Figure 2:**
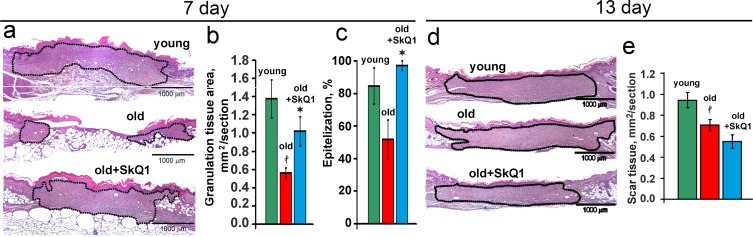
SkQ1 promotes granulation tissue formation and epithelization of old mice wound, but does not cause scar hypertrophy H&E staining of transverse sections of the wounds of old mice at 7d (**a**) and at 13d (**d**); area of granulation tissue or scar is shown by the black dotted line. (**b**) Granulation tissue formation, and (**c**) epithelization of the wounds at 7d; (**e**) scar formation at 13d. Data are presented as mean ± SD; *P < 0.05 for SkQ1-treated versus control; ‡P < 0.05 for the untreated young versus old mice.

**Figure 3:**
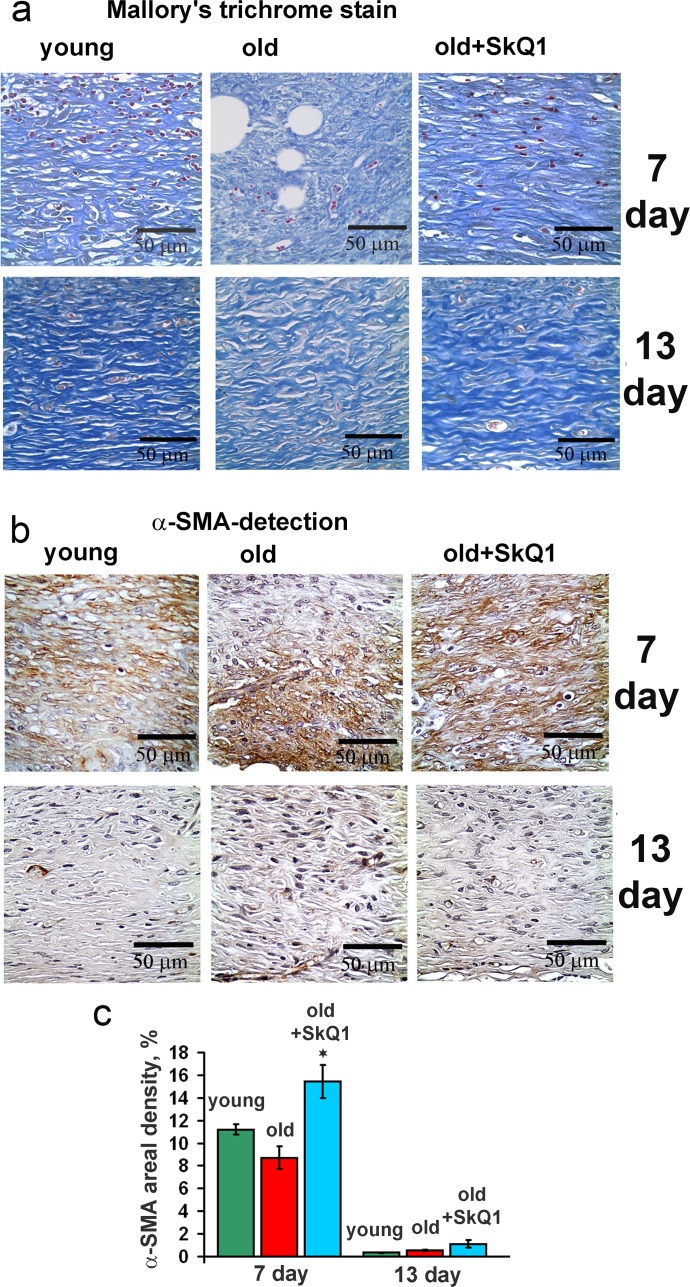
Effect of SkQ1 on the collagen fiber formation and α-SMA expression in granulation tissue and in scar (**a**) Mallory's trichrome staining and (**b**) α-SMA immunostaining of granulation tissue at 7 and 13 d. (**c**) Percentage of the area containing α-SMA-positive cells (areal density). Data are present-ed as mean ± SD; *P < 0.05 for SkQ1-treated versus control.

SkQ1 stimulated spreading of granulation tissue over the wounded area accelerating collagen fibers formation and maturation in old mice (Figs. 2, 3). Granulation tissue of SkQ1-treated mice contained increased amount of fibroblast-like cells expressing smooth muscle α-actin (α-SMA), (Fig. 3). Compared to fibroblasts, these cells referred to myofibroblasts produce more collagen, other extracellular matrix proteins and growth factors involved in granulation tissue formation and angiogenesis in wounds [35]. This finding was in perfect agreement with our earlier observations on SkQ1-induced myofibroblast differentiation of human subcutaneous fibroblasts *in vitro* [31].

In old mice wound epithelization was strongly compromised and SkQ1 significantly accelerated this process (Fig. 2). Such an effect was not accompanied by the increase of thickness in newly formed epithelium, so SkQ1 probably stimulated movement of epitheliocytes into the wound as it was observed earlier in the “wound” made in monolayer of epithelioid cells [32].

### SkQ1 accelerates resolution of inflammation

**Figure 4:**
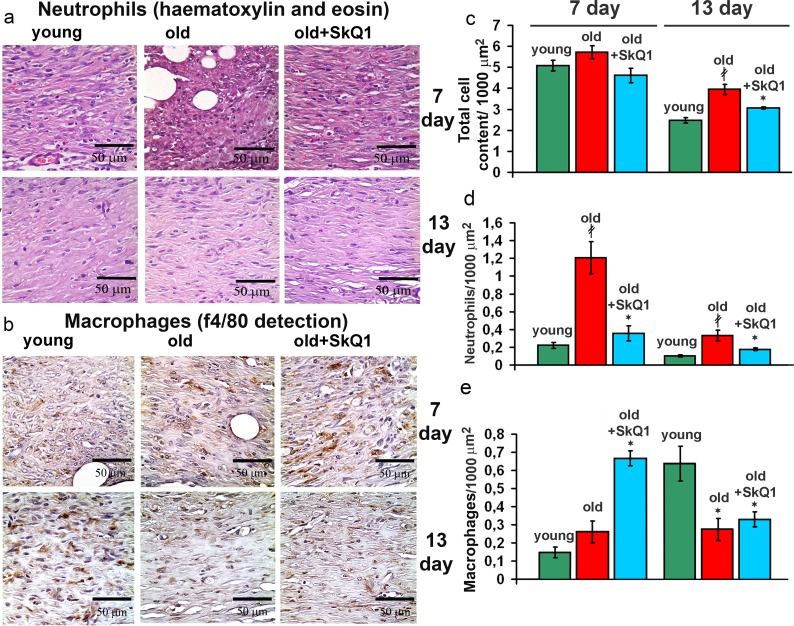
Effect of SkQ1 on the cellular composition of the wound (**a**) Representative micrographs of the H&E stained transverse sections at the edge of the wounds at 7d and 13d. (**b**) Edges of the wounds at 7d and 13d stained with antibodies against the macrophage marker F4/80. (**c**) Total cellular content, (**d**) infiltration of neutrophils and (**e**) macrophages (F4/80 positive cells) in granulation tissue at 7d and 13d. Data are presented as mean ± SD; *P < 0.05 for SkQ1-treated versus control; ‡P < 0.05 for the untreated young versus old mice.

Histological analysis revealed that old mice had significantly higher neutrophil content compared to the young animals at 7 day and even at 13 day after wounding. This phenomenon was described earlier for aged animals and humans [38, 39]. Treatment with SkQ1 strongly decreased the neutrophil infiltration in old mice (Fig. 4a, d). In parallel with increased neutrophil content, delayed infiltration of macrophages was observed in old mice while SkQ1 treatment accelerated this process (Fig. 4b, e). SkQ1 decreased macrophage content at 13 day to the level similar to that in young mice. These observations indicated that SkQ1 accelerated resolution of inflammatory phase.

**Figure 5:**
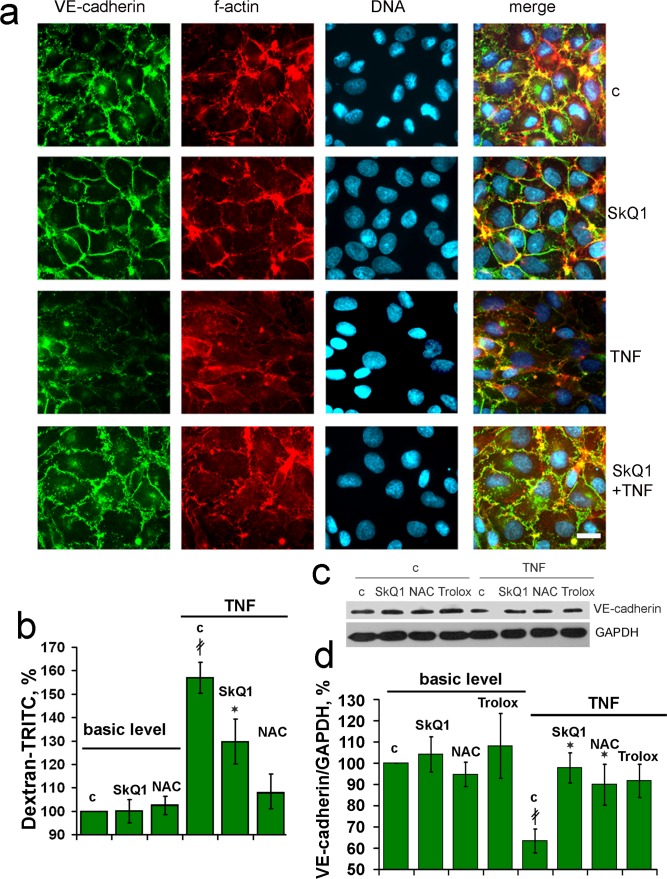
SkQ1 prevents TNF-induced decomposition of the endothelial cell-to-cell contacts containing VE-cadherin *in vitro* EA.hy926 cells were incubated for 4d with 20 nM SkQ1, 1 mM NAC or 0.1 mM Trolox and treated with 5 ng/ml TNF for 24h. (**a**) Detection of VE-cadherin (green), f-actin (red) and nuclei (blue). Bar, 15 μm. (**b**) Paracellular permeability assay in the cell monolayer using TRITC-dextran. (**c**) Representative immunoblotting of VE-cadherin, and (**d**) its quantification. “c” - untreated cells. Data are presented as mean ± SEM; N=4;*P < 0.05 for SkQ1+TNF-treated versus TNF treated samples; ‡P < 0.001 for the TNF treated versus untreated cells.

Compromised wound healing in old mice could be related to the increased inflammatory status. As we showed earlier, the levels of pro-inflammatory cytokines IL-6 and TNF in the blood were elevated in old animals and SkQ1 had no effect on these markers [11]. These data indicate that anti-inflammatory action of SkQ1 in the models of chronic wounds could be implemented in the tissues affected by an excessive inflammation. We have investigated the mechanisms of the possible anti-inflammatory action of SkQ1 in the culture of endothelial cells EA.hy926. These cells form typical epithelial monolayer with cell-to-cell contacts containing VE- cadherin (Fig. 5a).

Treatment of the monolayer with TNF(5ng/ml) induced significant disorganization of the VE-cadherin -containing contacts and actin filaments attached to the contact sites from cytosol. Pretreatment with SkQ1 protected the actin cytoskeleton, contacts and overall monolayer architecture against the TNF action (Fig. 5a). This effect correlated with the protection against TNF-induced increase in permeability of the monolayer for macromolecules (Fig. 5b). Decrease in VE-cadherin content in endothelium upon TNF treatment was described earlier [40, 41]. SkQ1 as well as non-targeted antioxidants NAC and Trolox inhibited this effect (Fig. 5c, d). As usual, non-targeted agent operated at very much higher concentration than SkQ1. Protection against TNF-induced decomposition of endothelial cell contacts could underlie inhibitory action of SkQ1 on transmigration and accumulation of neutrophils into the wound area.

### SkQ1 stimulates tissue remodeling

Excessive stimulation of myofibroblast differentiation may lead to the development of pathological scarring [36]. However, neither excessive accumulation of myofibroblasts nor other signs of fibrosis were detected in the scars at 13 days after wounding (Figs. 2d-e, 3). In fact, SkQ1 decreased cellularity of the scar tissue that was increased in old mice compared to young animals (Fig. 4c). These data indicated that tissue remodeling after wound closure was also accelerated by SkQ1. A full body histological examination of SkQ1-treated animals did not reveal any signs of fibrosis in lungs, liver and other organs.

### SkQ1 stimulates angiogenesis

**Figure 6:**
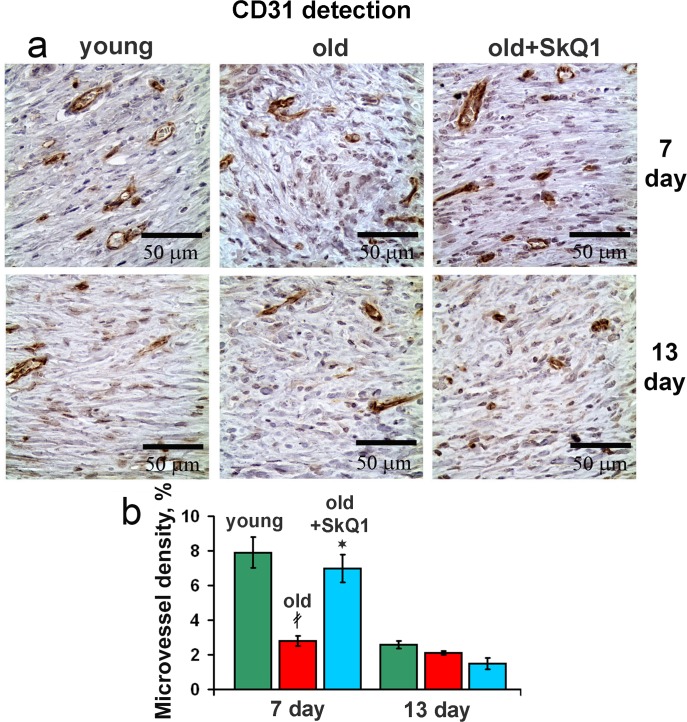
Effect of SkQ1 on the angiogenesis (**a**) CD31 immunostaining of wound area tissue at 7d and 13d. (**b**) Vessel density in CD31 stained sections of old mice. Data are presented as mean ± SD;*P < 0.05 for SkQ1-treated versus control; ‡P < 0.05 for the untreated young versus old mice.

**Figure 7:**
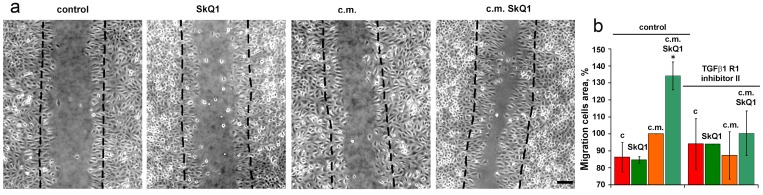
Effect of SkQ1 on EA.hy926 cell migration in scratch wound assay at 24 h The effect of 20 nM SkQ1, and conditioned medium (c.m.) from fibroblasts treated with 20 nM SkQ1. (**a**) Representative micrograph; bar, 60 μm. (**b**) Analysis of cell migration. The inhibitor of TGFβreceptor was added to scratch wound assay where indicated. Data are presented as mean ± SEM; N=4;*P < 0.05 for c.m. from SkQ1-treated versus untreated fibroblasts.

**Figure 8:**
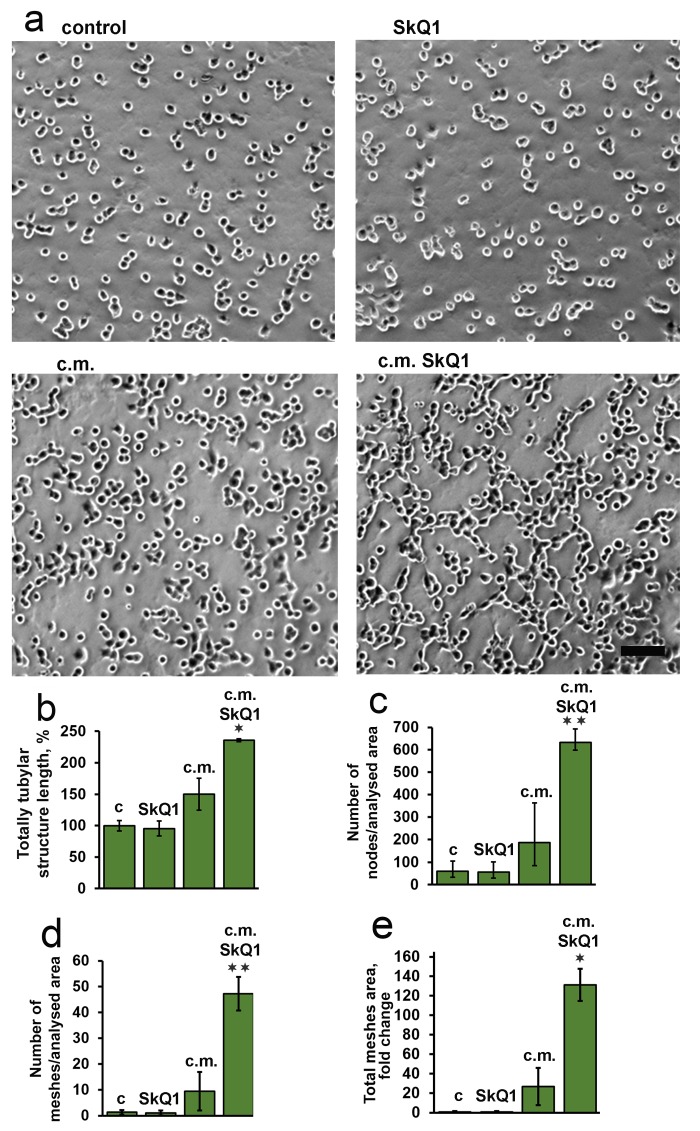
Effect of SkQ1 on endothelial EA.hy926 cell tubular structures formation on Matrigel The effect of 20 nM SkQ1, and conditioned medium (c.m.) from fibroblasts treated with 20 nM SkQ1. (**a**) Representative micrograph of matrigel angiogenesis assay; bar, 60 μm; (**b-e**) analysis of tube formation. Data are presented as mean ± SEM; N=5;*P < 0.05 **P < 0.001 for c.m. from SkQ1-treated versus untreated fibroblasts.

The volume density of microvessels in the granulation tissue of old mice was lower than in young animals indicating the delay in neovascularization. Treatment with SkQ1 increased the vessel content up to the normal value (Fig. 6). *In vitro* scratch-wound migration assay with endothelial EA.hy926 cells demonstrated that SkQ1 did not stimulate migration of endothelial cells, but the medium conditioned with SkQ1-treated human subcutaneous fibroblasts enhanced endothelial cells migration compared to the medium conditioned with untreated fibroblasts (Fig. 7). Earlier we have found that SkQ1 stimulated production of active TGFβ by fibroblasts [31], so we have tested the role of this cytokine in the effect of the conditioned medium. Inhibition of TGFβ receptor I by the specific inhibitor (TGFβR1 inhibitor II) partially prevented stimulation of cell migration by the conditioned medium (Fig. 7b). Angiogenesis also could be promoted by VEGF and other molecules produced by myofibroblasts, e.g. soluble mediators and/or microparticle's components [37]. The data of scratch-wound assay were consistent with the results of the endothelial cell tube formation assay. In a medium, containing reduced amount of growth factors (2% FBS) EA.hy926 cells did not form tubular structures on the surface of Matrigel and SkQ1 did not stimulate this process. At the same time, the conditioned medium from SkQ1-treated human subcutaneous fibroblasts stimulated migration-dependent processes, such as tubulogenesis, capillary growth, and sprouting (Fig. 8).

## DISCUSSION

Age-related disorders are listed among the main risk factors of impaired wound healing [1]. Wound healing is a complex process where three phases that overlap in time may be indicated: inflammation, granulation tissue formation, and tissue remodeling. All these phases are seriously compromised in the chronic wounds typical for aging. Prolonged treatment with mitochondria-targeted antioxidant SkQ1 significantly improved wound healing in old mice (Figs. 1-4). To our knowledge, this is the first report where such a dramatic acceleration of the wound healing process in old animals was achieved by any drug.

The excessive inflammation appears to be the ultimate cause of the poor wound healing in chronic wounds [42]. Neutrophil persistence and decreased macrophage infiltration is recognized as one of the crucial causes of chronic wounds in elderly patients [38, 39]. Treatment with SkQ1 resulted in the simultaneous decreased neutrophils and increased macrophages content in the wounds, indicating that resolution of the inflammatory phase was accelerated (Fig. 7). Earlier we have described the same effect of SkQ1 in dermal wounds and in the model of aseptic inflammation in young healthy mice [32].

It is widely accepted that the general inflammatory status increases with aging. Since SkQ1 had no effect on the level of circulatory IL-6 and TNF in old mice [11], we investigated putative anti-inflammatory action of SkQ1 in endothelium which is one of the most important targets for the pro-inflammatory cytokines. We have found that SkQ1 prevents TNF-induced disorganization of the actin cytoskeleton and VE-cadherin containing contacts, depletion of VE-cadherin from the cells and increase in permeability of the monolayer for macromolecules (Fig. 6). Earlier we have shown that SkQ1 decreased expression of the cell adhesion molecule ICAM1 in aortas of old mice and in endothelial cells treated with TNF via inhibition of NF-κB signaling [11]. SkQ1 also inhibited apoptosis of endothelial cells induced by high doses of TNF [43]. Our results both *in vivo* and *in vitro* indicate that mtROS could be critical for the complex response of the endothelium to the pro-inflammatory cytokines. Protection of the endothelium against overstimulation and following excessive migration of neutrophils into the wound could underlie the anti-inflammatory action of SkQ1. Prevention of excessive reaction of endothelium to the pro-inflammatory cytokines probably mediated not only anti-inflammatory effect of SkQ1 in impaired wound healing in old mice, but also therapeutic SkQ1 action in the animal models of various kidney, heart and brain pathologies [22, 23, 44]. SkQ1 stimulated formation of granulation tissue consisting of macrophages, fibroblasts and new capillaries in old mice (Figs. 2-4, 7). During morphogenesis of granulation tissue, various growth factors (especially TGFβ1) in concert with the ECM molecules stimulate fibroblasts to differentiate into myofibroblasts. The myofibroblasts produce TGFβ1, collagen and other ECM components, and participate in wound contraction [35, 45]. Earlier we have found that SkQ1 in the culture of subcutaneous fibroblasts stimulated TGFβ1 activation and following formation of α-SMA-positive cells (myofibroblasts) [31]. Scavenging of mtROS was shown to stimulate metalloprotease-dependent activation of latent TGFβ1 and downstream SMAD-dependent signaling.

Moreover, SkQ1 activated the Rho/ROCK/LIMK signaling pathway followed by phosphorylation of cofilin and stabilization of actin stress fibers [31]. We have observed that myofibroblast differentiation initiated by SkQ1 was mediated by autocrine stimulation of TGFβ1 but the fraction of α-SMA-positive cells never exceeded approx. 30% of the total cell population even during the prolonged (up to 3 months) treatment with SkQ1. This phenomenon was probably related to the ability of SkQ1 to attenuate myofibroblasts formation induced by excess of exogenous TGFβ1 [31]. This conclusion is in agreement with the recent data on the inhibition of TGFβ-dependent signaling by another mitochondria-targeted antioxidant MitoQ [46]. In perfect concert with these results, we have observed significant increase in α-SMA-positive cells content in the granulation tissue of SkQ1-treated animals (Fig. 3). Importantly, no excessive accumulation of myofibroblasts was observed at the late steps of wound healing in the scar tissue (Figs. 2, 3). Deregulation of ECM production by myofibroblasts may result in fibrosis and scarring [47]. Thorough histological examination of various tissues and organs of SkQ1-treated animals did not reveal any signs of fibrosis confirming that TGFβ1 activation and myofibroblast differentiation induced by SkQ1 remained under the pathological threshold.

Partial myofibroblasts differentiation induced by SkQ1 together with Rho-dependent stabilization of actin filaments contributed to acceleration of fibroblasts movement into the “wound” in the cell monolayer *in vitro* [32]. This effect could be important for improved formation of the granulation tissue and accumulation of myofibroblasts dus to it migration from the surrounding tissue or to fibroblast-to-myofibroblast differentiation in the wound. Another potential source of myofibroblasts are multipotent mesenchymal stromal cells (MSC) from the bone marrow [48]. Earlier we have found that prolonged treatment of mice with SkQ1 did not affect the number of MSC in the bone marrow but increased concentration of MSC progeny fibroblast colony forming units [49] so this source of myofibroblasts may also be activated by SkQ1.

During wound healing, TGFβ acts as a potent promoter of angiogenesis by stimulating cell proliferation, migration, capillary tube formation and deposition of extracellular matrix [50-52]. The *in vitro* experiments confirmed that TGFβ1 produced by the SkQ1-treated fibroblasts promoted movement (Fig. 7) and tubulogenesis (Fig. 8) of endothelial cells. In contrast to the endothelial cells, movement of the epitheliocytes into the *in vitro* “wound” was directly stimulated by SkQ1 [32] and this effect probably contributed to accelerated epithelization of the wounds in SkQ1-treated animals (Fig. 2).

Interestingly, age-related decline in dermal wound healing was strongly decelerated in the long-lived transgenic αMUPA mice. Such an effect phenotypicaly resembled the effects of caloric restriction (CR) [53]. These findings are consistent with the hypothesis on rejuvenation of wound-healing cells by the inhibitors of mTOR pathway, well known mimetics of CR [30, 54]. Excessive activation of mTOR was associated with vascular inflammation [55, 56] and mtROS may act as an important mTOR elicitor in a positive feedback loop [57].

In conclusion, our data demonstrate that the mitochondria-targeted antioxidant SkQ1 significantly accelerated both inflammatory and regenerative phases of wound healing in old mice. These effects were probably related to the inhibition of oxidative stress interfered with signaling pathways responsible for the resolution of inflammation and formation of granulation tissue. Our results pointed to endothelium and fibroblasts as the important targets for therapeutic action of the mitochondria-targeted antioxidants. These findings are in line with the numerous studies that demonstrated anti-aging effects and therapeutic action against age-related pathologies of SkQ1 and its analogs [58, 59].

## METHODS

All reagents used in this study were purchased from Sigma (Sigma, MO USA), unless otherwise indicated.

### Animals

24 month old F1 (CBAxC57Bl/6) mice (marked as “old”) received SkQ1 (100 nmol/kg of body weight per day) with drinking water during the last 8 month of life (n=10). The control groups consisted of 12 “old” and 12 “young” (6 month old) animals. All animal care and experimental procedures were conducted in compliance with European Directive-2010 of FELASA. Plasma concentrations of IL-6 and TNF were determined with ELISA kits (eBioscience, CA USA) according to manufacturer protocols.

Full-thickness 0.7 × 0.7-cm excisions of the skin were made in the interscapular area of the back and wound surface area was measured at the photographs using ImageJ software. Wounds and surrounding areas were incised and fixed with 10% phosphate-buffered formalin (pH 7.4), dehydrated and paraffin embedded. Cross-sections (5 μm thick) were stained with hematoxylin and eosin and Mallory's stain according to the routine protocols. For immunostaining, slices were treated with 3% H_2_O_2_ for 10 min, then with Antigen Unmasking Solution, high pH (Vector, CA USA) at 98°C, and incubated with polyclonal antibodies against α-SMA, CD31 (Abcam, UK) and monoclonal against f4/80 (Serotec, UK). Biotinylated anti-rabbit and anti-rat IgG were stained with avidin-peroxidase conjugate with diaminobenzidine (Vector). Images were analyzed with DM 5000B microscope (Leica, Germany) with DFC 320 digital camera. Neutrophils, macrophages and blood vessels were counted in 2*10^5^ μm^2^ of the granulation tissue.

### Cell cultures

Human subcutaneous fibroblasts HSF (Russian Cell Culture Collection, Institute of Medical Genetics, Russian Academy of Sciences) and endothelial cells EA.hy926 (ATCC^®^ CRL-2922) were cultured in DMEM (Gibco, MA USA) supplemented with 10% fetal calf serum (Hyclone, MA USA) and HAT in case of EA.hy926.

For immunostaining, Western blot and permeability assay EA.hy926 cells were incubated with 20 nM SkQ1, 1mM N-acetylcysteine (NAC) or 100 μM Trolox for 4 day and then treated with human recombinant TNF (5ng/ml, 48 hours).

For immunostaining cells were fixed with 2% paraformaldehyde, permeabilised with 0.2% Triton X-100 and incubated with phalloidin-TRITC, Hoechst 33342 and antibodies against VE-cadherin (eBioscience, CA USA). Secondary goat anti-mouse antibodies were conjugated with Alexa-488 (Molecular Probes, USA). Images were acquired using an Axiovert 200 microscope (Carl Zeiss, Germany).

VE-cadherin content was analyzed by Western blotting as described earlier [31] using antibodies against VE-cadherin (eBioscience, CA USA) and GAPDH (USBiological, MA USA). Goat antimouse antibodies conjugated with HRP were used as secondary antibodies. Membranes were developed with ECL chemilumines-cence reagents (Amersham, UK USA).

Permeability indicated by paracellular influx across the EA.hy926 monolayer was studied as described earlier [60]. Endothelial cells were seeded on Transwell filters (Corning Costar, NY USA). After antioxidant and TNF treatment, TRITC-labeled dextran tracer (1 mg/mL, 65-85 kDa) was added to the upper chamber. The amount of tracer penetrating through the cell monolayer into the lower chamber was measured fluorometrically.

Cell mobility was analyzed in scratch wound assay as described earlier [32]. EA.hy926 cells were treated with SkQ1 (20 nM, 4 days) and after wounding incubated with fresh or HSF conditioned medium. To collect the conditioned medium HSF were cultivated for 4 days with (or without) 20 nM SkQ1, washed and incubated for 48 h. Images were acquired using an Axiovert 200 microscope (Carl Zeiss, Germany) and processed by ImageJ 1.48.V software.

Endothelial cell tube formation was assayed as described earlier [61]. Control or SkQ1-treated (20 nM, 4 days) EA.hy926 cells were harvested with 0.125% trypsin-2 mM EDTA, resuspended in fresh growth factor-reduced medium (2% FBS) or HSF conditioned medium also containing only 2% FBS and plated in 24-wells plate (2 × 10^5^ cells/well) coated with Matrigel (10 mg/ml, 0.2 ml/well for 1 h at 37°C). Tubular structures were observed after 8 h of cell incubation at 37 °C and five randomly chosen fields of each well were acquired using an Axiovert 200 microscope (Carl Zeiss, Germany) and processed by Angiogenesis Analyzer macro written for ImageJ software (http://image.bio.methods.free.fr/ImageJ/?Angiogenesis-Analyzer-for-ImageJ.htm).

### Statistical analysis

Statistical analysis was done with STATISTICA 7.0 software. The data are expressed as mean ± SD or ± SEM (see figure legend). Student's unpaired t-test or Mann–Whitney U-test were conducted for comparisons, and significance was set at level p < 0.05.

## Abbreviations

mtROS: mitochondrial reactive oxygen species
SkQ1: 10-(6′-plastoquinonyl) decyltriphenylphosphonium
SkQR1: 10-(6′-plastoquinonyl) decylrhodamine 19
